# Co-delivery of paclitaxel and tetrandrine via iRGD peptide conjugated lipid-polymer hybrid nanoparticles overcome multidrug resistance in cancer cells

**DOI:** 10.1038/srep46057

**Published:** 2017-05-04

**Authors:** Jinming Zhang, Lu Wang, Hon Fai Chan, Wei Xie, Sheng Chen, Chengwei He, Yitao Wang, Meiwan Chen

**Affiliations:** 1 State Key Laboratory of Quality Research in Chinese Medicine, Institute of Chinese Medical Sciences, University of Macau, Macau, China; 2 School of Pharmacy, Chengdu University of Traditional Chinese Medicine, Chengdu 611137, China; 3 Department of Biomedical Engineering, Columbia University, New York, NY 10027, USA; 4 Department of Respiratory Medicine, Shenzhen Traditional Chinese Medicine Hospital, Shenzhen 518033, China

## Abstract

One of the promising strategies to overcome tumor multidrug resistance (MDR) is to deliver anticancer drug along with P-glycoprotein (P-gp) inhibitor simultaneously. To enhance the cancer cellular internalization and implement the controlled drug release, herein an iRGD peptide-modified lipid-polymer hybrid nanosystem (LPN) was fabricated to coload paclitaxel (PTX) and tetrandrine (TET) at a precise combination ratio. In this co-delivery system, PTX was covalently conjugated to poly (D,L-lactide-co-glycolide) polymeric core by redox-sensitive disulfide bond, while TET was physically capsulated spontaneously for the aim to suppress P-gp in advance by the earlier released TET in cancer cells. As a result, the PTX+TET/iRGD LPNs with a core-shell structure possessed high drug loading efficiency, stability and redox-sensitive drug release profiles. Owing to the enhanced cellular uptake and P-gp suppression mediated by TET, significantly more PTX accumulated in A2780/PTX cells treated with PTX+TET/iRGD LPNs than either free drugs or non-iRGD modified LPNs. As expected, PTX+TET/iRGD LPNs presented the highest cytotoxicity against A2780/PTX cells and effectively promoted ROS production, enhanced apoptosis and cell cycle arrests particularly. Taken together, the co-delivery system demonstrated great promise as potential treatment for MDR-related tumors based on the synergistic effects of P-gp inhibition, enhanced endocytosis and intracellular sequentially drug release.

Ovarian cancer exhibits the highest mortality rate among all gynecological cancers worldwide^1^. Chemotherapy with paclitaxel (PTX), a mitosis-disrupting agent, is currently the standard ovarian cancer treatment in clinic. However, the frequent occurrence of multidrug resistance (MDR) is a common obstacle to effective chemotherapy, leading to chemotherapy failure in the majority of ovarian cancer patients. The molecular mechanism of PTX resistance in ovarian cancer cells is related to the overexpression of P-glycoprotein (P-gp), an ATP-binding cassette transporter, which results in PTX cellular efflux^2^. About 40–50% of patients are found with up to 100-fold P-gp overexpression in their tumor tissues compared to the normal tissues. The intracellular concentration of PTX was decreased in the presence of P-gp which led to a reduction of anticancer efficacy in cancer cells. Accordingly, the coadministration of P-gp inhibitors with anticancer drugs could efficiently overcome efflux-mediated drug resistance^3^. Combination therapy of anticancer drugs and P-gp inhibitors has been demonstrated to decrease tumor volumes and prolong lifespans in preclinical studies^4^.

The P-gp inhibitor, tetrandrin (TET) which is a bis-benzylisoquinoline alkaloid isolated from the root of *Stephania tetrandra* S. Moore, exhibits potent reversal of MDR by directly binding to P-gp and increasing intracellular drug accumulation^5^. Moreover, compared to the common P-gp inhibitors, TET demonstrates higher level of antitumor activities by suppressing cancer cell proliferation, inducing apoptosis and autophagy, and promoting reactive oxygen species (ROS) production^6^,^7^,^8^. Previous studies have confirmed the synergistic anticancer activities of clinical anticancer drugs such as sorafenib^9^, cisplatin^10^, 5-fluorouracil^11^ in combination with TET. Li’s group^12^ revealed that the combination of PTX and TET effectively induced the apoptosis of gastric cancer cells by an enhanced intracellular oxidation mechanism. The MDR-reversing activity of TET combined with PTX has been identified^13^, as 2.5 μM of TET could reverse the sensitivity of KBv200 cells to PTX by 10-fold and potentiate the antitumor activity of PTX in xenograft models bearing intrinsically resistant KBv200 tumors. Thus, the combination of PTX with TET is a promising strategy for overcoming MDR and improving anticancer effects.

Although combination therapy is regarded as a potential cancer treatment regimen in clinic, its current therapeutic outcomes are far from perfect^14^. The different pharmacokinetics, biodistribution and membrane transport properties of various anticancer drugs and P-gp inhibitors result in inconsistent *in vivo* circulation and cellular uptake profiles for different agents. The suboptimal drug combinations at the tumor site would compromise the synergetic effects provided by combination treatment^15^. Hence, the encapsulation of multiple drug cargoes within a single nanocarrier could facilitate the simultaneous delivery of drugs at a precise combination ratio at the tumor site^16^,^17^. Co-delivery of MDR-related anticancer drugs and P-gp modulators using nanocarrier strategy has demonstrated colocalization of more drugs in tumor tissue compared to free drug combinations^18^,^19^,^20^.

Moreover, the decoration of targeting ligands on nanoparticles (NPs) to recognize specific receptors overexpressed on tumor cell surface can maximize drug delivery to the tumor site and effectively overcome tumor drug resistance^21^,^22^. iRGD (CRGDK/RGPD/EC), a tumor-homing peptide, has been shown to enhance the delivery of compounds or NPs into the extravascular tumor parenchyma^23^,^24^. The peptide’s active recognition of αvβ_3/5_ integrin receptors helps to improve drug delivery using PEGylated NPs that is characterized by long blood circulation and poor drug uptake. Furthermore, we hypothesize that in the process of co-delivery of multiple drugs, when the P-gp inhibitors are released earlier and faster than the anticancer drugs to suppress drug efflux pumps, fewer MDR-related drugs would be ejected out. This approach could dramatically improve anticancer drug accumulation in tumor cells^25^. Nevertheless, most of the current studies on co-delivery strategies suffer from limitations^26^,^27^,^28^. In most cases, multiple drugs were simultaneously encapsulated in a single nanocarrier based on the interaction between the drugs and hydrophobic polymeric cores. The competition between multiple drugs often leads to low drug loading efficiencies and NP instabilities^29^. On the other hand, polymer-drug conjugates have extensively been employed as prodrugs and significantly reduced the intermolecular competition of drugs^30^. The co-delivery of polymer-drug conjugates with small molecule drugs could eliminate the issue of low drug loading efficiency due to intermolecular interactions between free drugs, batch-to-batch variability in drug loading and release kinetics.

In this study, an iRGD-mediated lipid-polymer hybrid nanosystem (LPN)^31^ was designed to co-deliver PTX and TET at a precise optimized drug ratio to overcome MDR in ovarian cancer. To reduce the instability resulting from the interactions between different drug molecules, PTX was first conjugated to poly (lactic-co-glycolic acid) polymer via disulfide linkages to serve as the core of the LPN. PTX would then be released into the cytoplasm via the breakage of disulfide bonds (10 mM glutathione)^32^. Subsequently, TET was loaded into the LPN by a single-step method involving nanoprecipitation and self-assembly process. This novel strategy combines the following advantages: (1) tumor targeting and penetration mediated by the iRGD peptide; (2) combined intracellular uptake of PTX and TET; (3) improved cytotoxicity against MDR cancer cells by the temporal drug release profiles. Our study aimed to demonstrate that the targeted LPN system delivering a combination of an anticancer drug and P-gp inhibitor in a temporal drug release manner could effectively overcome tumor drug resistance.

## Results

### Synthesis and characterization of PLGA-SS-PTX conjugate

**Figure 1:**
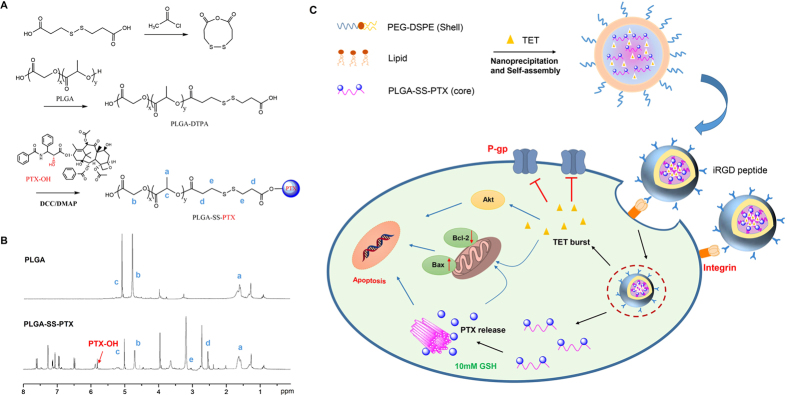
(**A**) Synthesis route of PLGA-SS-PTX conjugate; **(B)** ^1^H NMR spectra of PLGA polymer and PLGA-SS-PTX conjugate. (**C**) scheme image of MDR reversal by PTX and TET co-delivery in iRGD peptide conjugated NPs.

PTX was conveniently conjugated to PLGA polymer with a disulfide linkage via two synthesis steps, as shown in Fig. 1A. DTPAA was first synthesized to replace DTPA, which is commonly used as a disulfide linkage, to effectively avoid the generation of PLGA-SS-PLGA. The chemical structure of the resulting PLGA-SS-PTX conjugate was verified by ^1^H NMR analysis (Fig. 1B). The main characteristic resonances of PLGA and PLGA-SS-PTX were identified. In addition, the typical methylene peaks of DTPA (-CH_2_-CH_2_-S-S-CH_2_-CH_2_-) at δ 2.52 ppm and δ 3.05 ppm were identified in PLGA-SS-PTX, indicating the successful conjugation between PLGA and DTPA. Similar to results published in a previous literature report, the characteristic resonance of active -CH proton of conjugated PTX shifted from about 4.6 ppm to 5.82 ppm, indicating that the esterification reaction between the active hydroxyl of PTX and PLGA-DTPA occurred preferentially^33^.

After maintained in hydrochloric acid (1 M) for 30 min at 60 °C, conjugated PTX was cleaved from PLGA-SS-PTX. A 12.6% LE of PTX in PLGA-SS-PTX was determined by HPLC analysis. Our result was opportune in accordance with the molar ratio (1/1) of PLGA and PTX in this conjugate. Such a high LE is not easy to obtain in most common NPs as most of previous reports demonstrated LEs of lower than 5%^34^.

### *In vitro* combination effect of free PTX and TET

The *in vitro* cytotoxicity studies were first conducted with various PTX/TET combinations in A2780/PTX cells to determine whether TET would reverse drug resistance and identify the optimum ratio of PTX/TET with a strong synergistic effect. The results were shown in Table 1. Compared to PTX drug sensitive A2780 cells, A2780/PTX cells demonstrated high drug resistance against PTX, with a 48.5-fold increase in the IC_50_. All combination groups were able to sensitize A2780/PTX cells to PTX cytotoxicity. The reversal indexes ranged from 3–8 when TET was used at different ratios with PTX. Furthermore, the CI values for the IC_50_ were much less than 1, which indicated an obvious synergistic effect between PTX and TET. Since the lowest CI value for the PTX/TET combination was obtained at a 1/1 molar ratio, this combination was chosen as the ideal synergistic ratio for the following experiments.

### Preparation and characterization of PTX+TET/iRGD LPN

**Figure 2:**
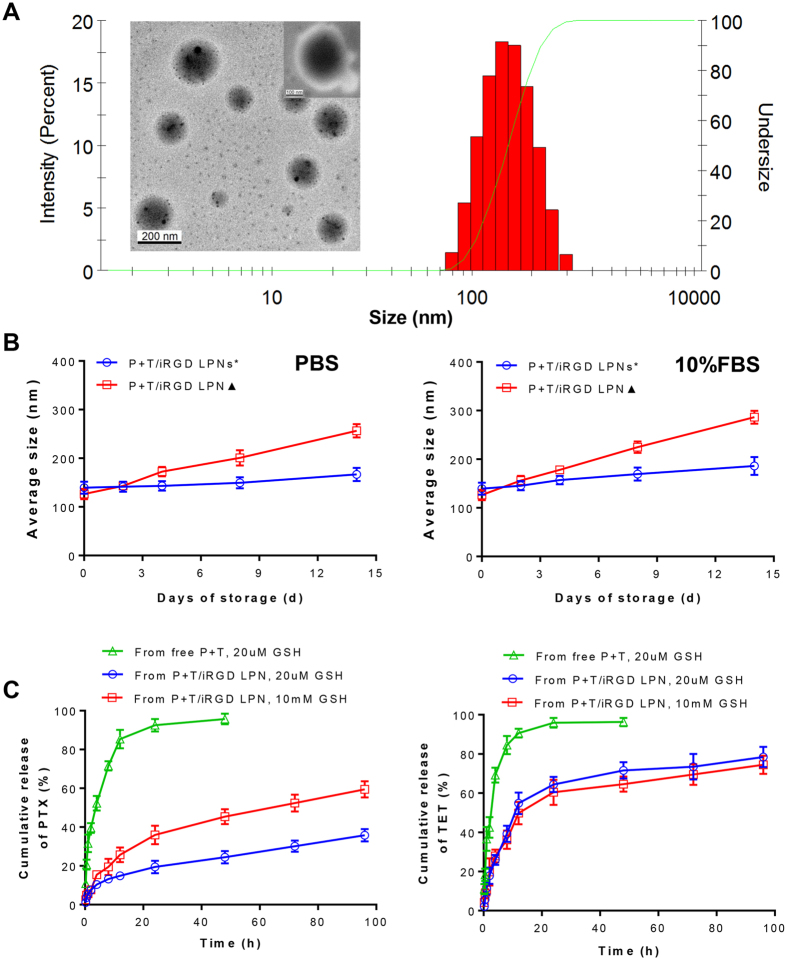
Characterization of PTX and TET co-loaded LPN. **(A)** Particle size distribution measured by DLS and TEM image of LPN. (Top-right) The magnified view of LPN showing the core-lipid shell structure; **(B)** Stability of conjugated P+T/iRGD LPN^*^ and unconjugated P+T/iRGD LPN^▲^ in PBS or 10% FBS for 14 days storage; **(C)** *in vitro* release of PTX and TET in response to redox environment.

The PTX and TET co-loaded LPNs with a core-shell lipid-polymeric structure and iRGD peptides on the surface were formulated by nanoprecipitation and self-assembly (Fig. 1C). P/LPNs with or without iRGD peptide decoration were prepared as controls in the following experiments by a similar procedure without TET feeding. To achieve the optimized combination ratio of PTX/TET, the amounts of drug input were screened for optimal physical properties. As shown in Table 2, all of these prepared nanoformulations exhibited a favorable particle size (less than 150 nm), negative zeta potential and high EE or LE of either PTX or TET. No significant alterations in size, zeta potential or LE were observed with or without iRGD peptide decoration. The DLS spectrum and TEM images of the PTX+TET/ iRGD LPNs were shown in Fig. 2A. Specifically, to demonstrate the effect of polymer-drug conjugation on stability, both PLGA-SS-PTX conjugates and the unconjugated PLGA polymers were utilized to fabricated two types of LPN, i.e. P+T/LPN* and PTX+TET/LPN^▲^, respectively. Over the course of two weeks, the size of the P+T/LPN^▲^ exhibited greater variation than the conjugated P+T/LPN* in both PBS and 10% FBS buffer (Fig. 2B), which suggested that weaker interactions between drugs due to polymer-drug conjugation could improve particle stability.

### *In vitro* drug release study

Both PTX and TET in the LPNs exhibited sustained release profiles in medium containing 20 μM GSH, compared with the free drug combinations (Fig. 2C). Furthermore, due to its conjugation to the PLGA polymer, PTX demonstrated slower release behavior from the LPNs than TET at 20 μM GSH. Interestingly, the PTX in the LPNs displayed a GSH-dependent release profile. When the LPNs were placed in medium containing 10 mM GSH, which was close to the GSH concentration found in cancer cells, the PTX in the LPNs was released faster in response to the highly reducing environment. Nevertheless, the total accumulation of PTX was still less than that of TET during the same time period, which exactly confirmed our hypothesis about the programmed drug release behavior.

### Cellular uptake of PTX

**Figure 3:**
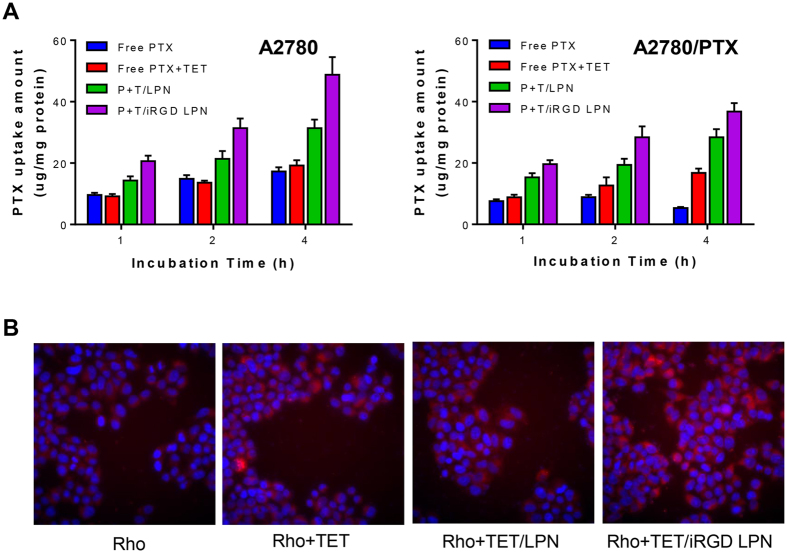
(**A**) Intracellular uptake of PTX of free PTX, PTX+TET, P+T/LPN, and P+T/iRGD LPN in A2780 and A2780/PTX cells after 1 h, 2 h, and 4 h incubation. (Various groups *vs* free P+T ^#^p < 0.05, P+T/LPN *vs* P+T/iRGD LPN ^‡^p < 0.05). **(B)** Rhodamine 123 accumulation of free Rho 123, Rho 123+TET, R+T/LPN, and R + T/iRGD LPN in A2780/PTX cells for 4 h pretreatment and 1 h efflux. Scale bar is 50 μm.

The cellular PTX accumulation in both sensitive A2780 and nonsenstive A2780/PTX cells was further evaluated. The accumulation of PTX in various treated cells was time-dependent (Fig. 3A). However, the inclusion of TET did not impact the cellular uptake of PTX in A2780 cells, whereas it did enhance the PTX concentration in A2780/PTX cells due to the suppression of efflux activity. The total accumulation of P+T/iRGD LPNs in both A2780 and A2780/PTX cells was higher than that of the other formulations at each time interval. The highest cellular uptake of the P+T/iRGD LPNs in A2780/PTX cells indicated the synergetic effects of P-gp inhibition by TET and the iRGD-mediated uptake of nanocarriers.

### Intracellular accumulation of Rhodamine 123 in A2780/PTX cells

The fluorescent agent Rho 123, a P-gp efflux substrate, was used to replace PTX and to visualize the intracellular accumulation of the LPNs. Rho 123 was conjugated to PLGA polymer and co-loaded with TET into LPNs. After various LPNs were incubated in A2780/PTX cells for 1 h, the amounts of Rho 123 accumulating in cells after various treatments were shown in Fig. 3B. The cells pretreated with iRGD LPNs containing co-loaded Rho 123 and TET displayed the highest level of red fluorescence, suggesting the greatest amount of Rho 123 accumulated in A2780/PTX cells due to the combined effect of uptake and efflux inhibition with TET/iRGD LPNs. This finding aligned with the quantitative results obtained for intracellular PTX accumulation.

### P-gp inhibition and cytotoxicity study

**Figure 4:**
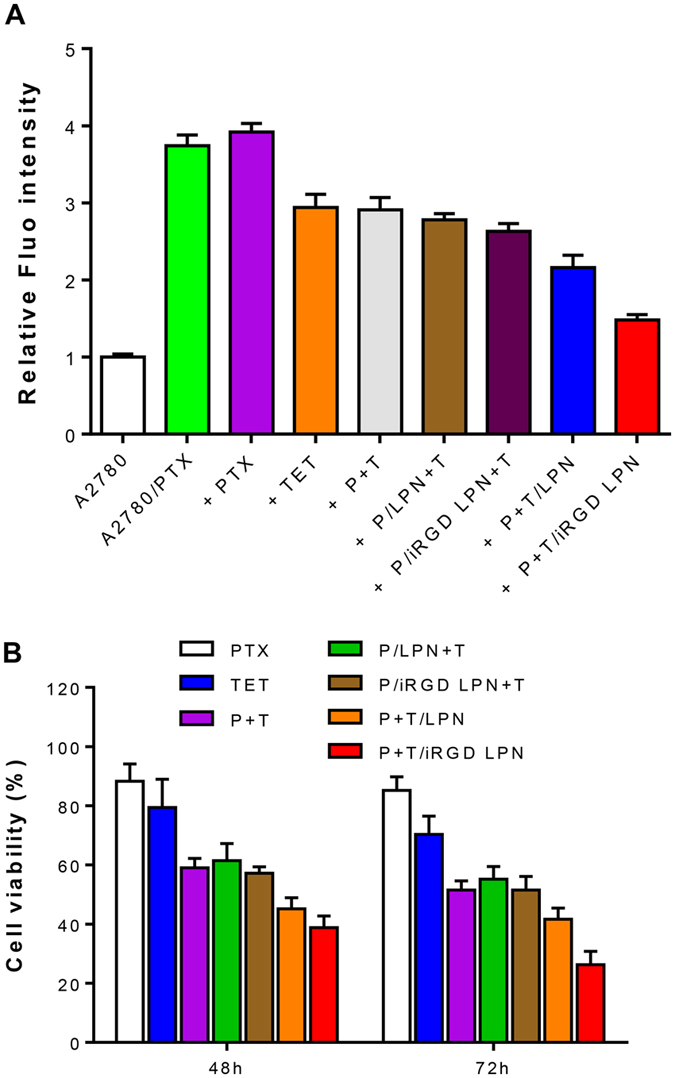
(**A**) P-gp inhibition effects of various formulations on A2780/PTX cells. **(B)** Cytotoxicity against A2780/PTX cells of various formulations including free PTX, free TET, PTX+TET, P/LPN+T, P/iRGD LPN+T, P+T/LPN, and P+T/iRGD LPN with equivalent concentrations of both PTX and TET at 10 μM for 48 h and 72 h. (A2780 *vs* A2780/PTX cells ^▲^p < 0.05, free PTX *vs* P+T ^†^p < 0.05, *vs* free P+T ^#^p < 0.05, P+T/LPN *vs* P+T/iRGD LPN ^‡^p < 0.05.)

The P-gp expression of A2780 or A2780/PTX cells after pretreatment with various formulations was detected by flow cytometry analysis (Fig. 4A). Remarkably higher P-gp expression was observed in A2780/PTX cells than in A2780 cells. In the following experiment, the A2780/PTX cells were pretreated with free drug or nanoparticle combinations for 4 h. All formulations containing TET generated different degrees of P-gp inhibition, with P+T/iRGD LPNs exhibiting the most potent efficacy in suppressing P-gp activity. Interestingly, P/LPN+T, the mixture of LPNs containing only PTX and free TET, was similarly effective compared to the free P+T treatment. The result demonstrated the significance of encapsulating PTX with a P-gp inhibitor in a single carrier.

Moreover, based on the IC_50_ value of free P+T in A2780/PTX cells, 10 μM concentrations of PTX and TET were chosen as the optimal value for the following experiments. The cells were treated with blank LPNs at 25–500 μg/mL concentrations for 72 h. Above 90% cell viability was observed, indicating that the blank LPNs possessed high biocompatibility. Subsequently, the cytotoxicity of various formulations in A2780/PTX cells after 48 h and 72 h incubations were determined by cell proliferation analysis (Fig. 4B). Similar to the P-gp inhibition results, the level of cytotoxicity among free P+T, P/LPN+T, and P/iRGD LPN+T was comparable. Importantly, the P+T/iRGD LPNs exhibited much higher level of cytotoxicity than P+T LPNs or free P+T treatments, indicating the synergistic antiproliferative effect of PTX and TET co-loaded in iRGD-decorated nanoparticles.

### Intracellular ROS level determination

**Figure 5:**
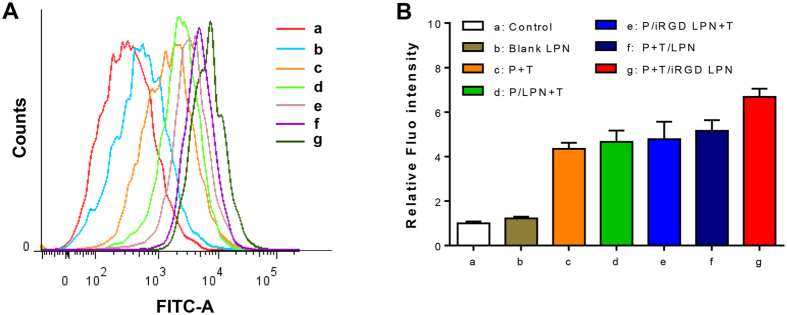
Intracellular ROS levels induced by various formulations including untreated control (**a**), blank LPN (**b**), PTX+TET(**c**), P/LPN+T (**d**), P/iRGD LPN+T (**e**), P+T/LPN (**f**), and P+T/iRGD LPN (**g**) with equivalent concentrations of both PTX and TET at 10 μM for 24 h measured by FCM **(A)** and quantified by relative fluorescence intensity **(B)**. (Various groups *vs* untreated control ^#^p < 0.05, P+T/LPN *vs* P+T/iRGD LPN ^‡^p < 0.05).

The ROS levels in various pretreated A2780/PTX cells were determined by measuring the generated green fluorescence intensity using FCM. The FCM data were analyzed using FlowJo 7.6 software (Fig. 5A). The ROS levels in the different cell treatment groups were shown in Fig. 5B. Blank LPNs exerted no influence on the intracellular ROS levels, which were as low as that ofthe untreated controls. However, the free P+T, P/LPN+T, P/iRGD LPN+T, and P+T/LPN treatments induced increased ROS levels in the A2780/PTX cells, compared to the untreated controls. According to a previous report^35^, an acceleration in ROS production could promote apoptosis. As an apoptosis inducer, the TET increased the levels of ROS. Most importantly, the co-delivery of PTX and TET in the iRGD LPNs triggered significantly more ROS production in the cells, compared to the other groups. The results indicated that P+T/iRGD LPN promoted apoptosis activity in A2780/PTX cells.

### Apoptosis analysis by Annexin V-FITC/PI staining

**Figure 6:**
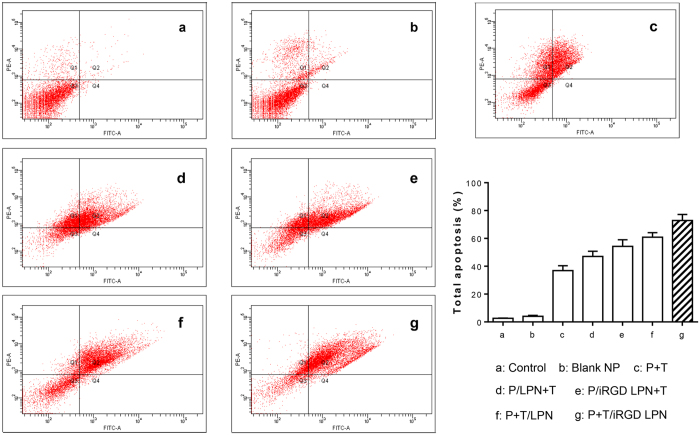
Apoptosis level induced by various formulations including untreated control, blank LPN, PTX+TET, P/LPN+T, P/iRGD LPN+T, P+T/LPN, and P+T/iRGD LPN with equivalent concentrations of both PTX and TET at 10 μM for 48 h. (Various groups *vs* free P+T ^#^p < 0.05, P+T/LPN *vs* P+T/iRGD LPN ^‡^p < 0.05).

Apoptosis analysis by the Annexin V-FITC/PI staining method was further used to confirm the proapoptotic effects of P+T/iRGD LPNs treatment. These formulations with drugs loaded in LPNs generated higher apoptosis rates than the free P+T combination (Fig. 6), indicating the advantages of utilizing iRGD on LPNs and the co-delivery of drugs. Interestingly, a higher apoptosis rate for the P/LPN+T and P/iRGD LPN+T groups, compared to the free P+T group, was observed. This might have resulted from the enhanced PTX uptake of PTX incorporated in the LPNs compared with free PTX. However, the P+T/iRGD LPN treatment generated a 70% of total apoptosis rate after 48 h incubation, which was higher than that of either the P+T/LPN or non-coloaded LPNs groups. Although P-gp inhibitors have been combined with anticancer drugs to promote cytotoxicity previously^36^, the efficacy of co-delivery of drugs in a single carrier was much superior.

### Cell cycle analysis

**Figure 7:**
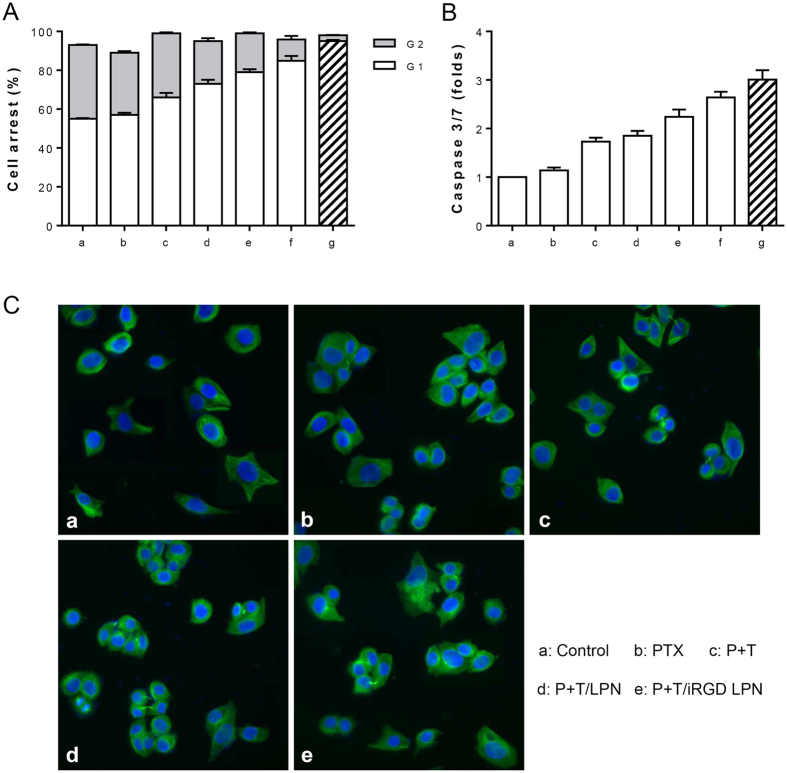
Cell cycle distribution **(A)** and caspase 3/7 activity **(B)** of A2780/PTX cells treated by various formulations including untreated control (a), blank LPN (b), PTX+TET(c), P/LPN+T (d), P/iRGD LPN+T (e), P+T/LPN (f), and P+T/iRGD LPN (g) with equivalent concentrations of both PTX and TET at 10 μM after 48 h incubation. (Various groups *vs* free P+T ^#^p < 0.05, P+T/LPN *vs* P+T/iRGD LPN ^‡^p < 0.05.) **(C)** Tubulin immunofluorescence images of A2780/PTX cells treated by various formulations including free PTX, PTX+TET, P+T/LPN, and P+T/iRGD LPN with equivalent concentrations of both PTX and TET at 10 μM for 24 h.

The cell cycle distribution of the treated A2780/PTX cells was sequentially investigated by FCM. The biocompatibility of blank LPNs and the G1 phase arrest after the application of other formulations were observed (Fig. 7A). While PTX was confirmed to block the cell cycle at the G2/M phase, TET was demonstrated to disturb many cancer cells at the G1 phase^6^,^37^. The inclusion of TET blocked the cell cycle at the G1 phase in advance, so any G2-phase arrest might not happen. Indeed, since TET in the co-loaded LPNs was released earlier than the PTX, the combination of PTX and TET generated G1 phase arrest. Likewise, P+T/iRGD LPN-treated cells exhibited significantly higher cell cycle arrest at the G1 phase than its treatment counterparts. Overall, our result demonstrated that the enhanced cytotoxicity generated by TET co-loaded was partly caused by the cell cycle blocking activity of TET.

### Caspase-3/7 determination

The activity of caspase-3/7 which is essential for apoptosis in various treated A2780/PTX cells was determined. Compared to untreated control, exposure of cells to various drug formulations for 48 h treatment remarkably raised the caspase-3/7 activity. Only a slight increase in caspase-3/7 activity was observed in cells treated with free P+T. After P+T/iRGD LPN treatment, the caspase-3/7 activity was elevated about three-fold compared to the control (Fig. 7B). Our data provided more support for the potent proapoptotic effects of P+T/iRGD LPNs.

### Tubulin immunofluorescence analysis

As the main hallmark of PTX, tubulin polymerization in various treated cells was evaluated by immunofluorescence^38^. In untreated cells, a well-organized tubulin network with green fluorescence was observed, whereas more green fluorescence dots were observed around the nucleus in cells exposed to formulations containing PTX (Fig. 7C). With P+T/iRGD LPN treatment, most of the punctate bright fluorescent spots of tubulin were observed in close proximity to nucleus in many cells. This finding revealed that P+T/iRGD LPNs obviously promoted the polymerization of tubulin, which could block cancer cell mitosis.

### Apoptosis-related protein expression

**Figure 8:**
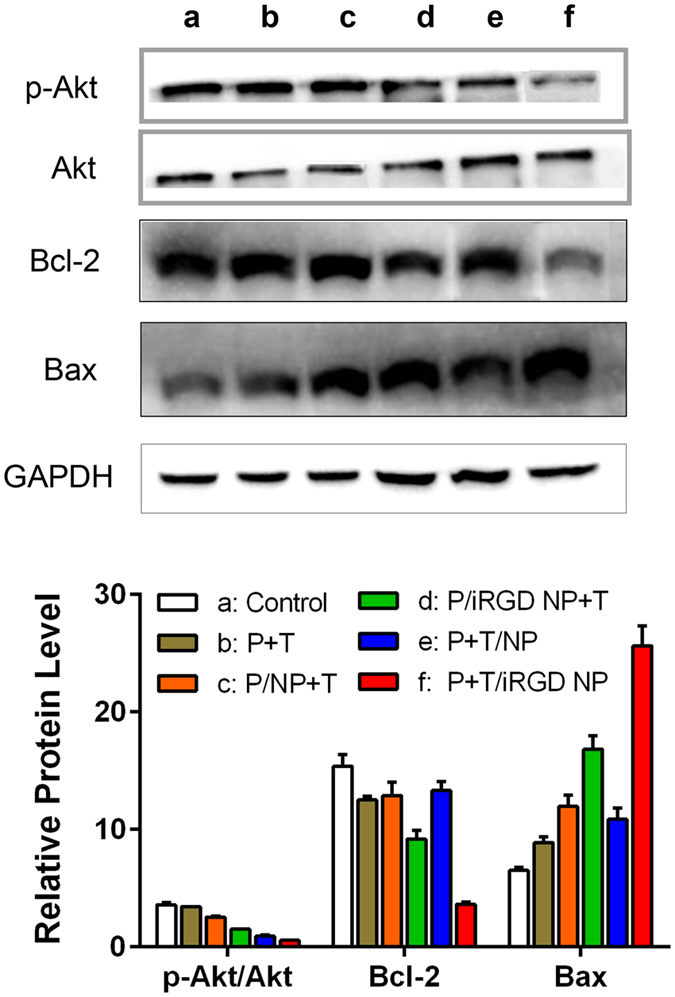
Effects of various formulations with PTX and TET at 10 μM concentration on the expression of apoptotic proteins in A2780/PTX cells after 24 h treatment. Upper panel: Western blot analysis of p-Akt, total Akt, Bcl-2, and Bax in cells exposed to various agents. Lower panel: semi-quantitative analysis of protein in different groups. (Various groups *vs* free P+T ^#^p < 0.05, P+T/LPN *vs* P+T/iRGD LPN ^‡^p < 0.05).

Given the significant intracellular ROS induction activity of P+T/iRGD LPN, its effect on the apoptotic pathway related to Akt, Bcl-2, and Bax proteins was also evaluated by western blotting analysis (Fig. 8). After 24 h incubation, compared to untreated control, all formulations resulted in a decrease of p-AKt, relative stability of Akt, a decrease of Bcl-2 and an increase of Bax. Quantification of the activities of these proteins revealed the most significantly change of activities was observed in cells treated with P+T/iRGD LPNs. These iRGD co-loading NPs possessed significantly stronger capacity to regulate apoptosis pathway, compared to either free P+T or non-iRGD NPs (p < 0.05). Furthermore, P+T/iRGD LPNs statistically surpassed the combination of PTX NPs and free TET, which was in line with the cytotoxicity and apoptosis results above-mentioned. The apoptosis-related protein expression results indicated that to co-delivery of PTX and TET in iRGD peptide decorated NPs may be a more effective strategy to overcome MDR in ovarian cancer.

## Discussion

Since PTX was approved by the FDA in 1992 for the treatment of ovarian cancer, PTX has been widely used in clinic as an anticancer drug. Although PTX application has improved the duration and quality of life for some ovarian cancer patients, unfortunately, the majority eventually suffer from the attenuated therapeutic outcome owing to drug resistance as in the case of other chemotherapeutic drugs. Therefore, drug resistance represents a major obstacle to improving the overall response and survival of cancer patients. A wide variety of drug resistance mechanisms was uncovered, such as overexpression of multidrug resistance (MDR-1) gene (P-gp), molecular changes in microtubule, changes in apoptotic regulatory and mitosis checkpoint proteins^39^,^40^. Thus, in this study, the P-gp inhibitor (TET) was combined with PTX to overcome the drug resistance. Although the synergetic effect of TET and PTX against the resistant KBv200 cells was previously reported^13^, their effect in ovarian cells is still unclear. The combination ratios for the synergetic MDR overcoming effect were optimized in Table 1. Our results indicated that combining PTX and TET at 1/1 molar ratio would translate into the best combination activity and over 5-fold of reversal efficiency. It provides favorable evidence to overcome MDR.

To directly co-deliver multiple drugs in nanoparticles by one-step self-assembly or nanoprecipitation technology, several problems are usually encountered such as the random amount of loaded drug and low loading efficiency due to intermolecular interactions. A number of studies have addressed these issues via producing temporal co-loading^5^,^41^,^42^ or multi-layered NPs^43^,^44^. Particularly, to maximize the suppression of the drug efflux, these P-gp inhibitors should be released earlier in cell than PTX so that higher amount of PTX could accumulate intracellularly. In our study, we fabricated a co-loaded LPN with a sequential drug-release profile, where PTX was conjugated to the PLGA polymer core by the disulfide linkages and TET was encapsulated in hydrophobic polymeric core. In this approach, intracellular TET release will precede that of PTX. Subsequently PTX will be released in cytoplasm in the presence of higher GSH concentration (10 mM). The time-dependent sequential release of these two drugs was demonstrated in Fig. 2C. Less than 60% of PTX from P+T/iRGD LPN accumulated in 10 mM GSH buffer during 96 h treatment. Nevertheless, over 60% of total TET from P+T/iRGD LPN were detected cumulatively at 24 h. The sequential release profile is beneficial to temporally overcoming MDR and improving anticancer efficacy.

iRGD peptide is frequently utilized to improve the tumor penetration effects of nano-carriers^45^,^46^. To achieve the enhanced tumor targeting efficacy, the substantial decoration of iRGD on nano-carriers is essential. Shen *et al*.^46^ conjugated iRGD peptide on the TPGS polymer by the reaction between acrylolyl group and amino group. Similarly, iRGD-conjugated nanoparticles were prepared though a thiazolidine ring^47^. Complex purification procedures were involved in these reports. In this study, iRGD peptide was covalently coupled with LPN surface using the post-insertion technique via a mild maleimide-thiol coupling reaction. The coupling efficiency of iRGD could be conveniently detected via the iRGD quantification of HPLC. This method has been widely applied in the modification of tumor targeting liposomes^48^.

As expected, PTX and TET coloaded iRGD LPN (P+T/iRGD LPN) exhibited higher cytotoxicity than free drug combination or non-targeted LPN as shown in Fig. 4B. Our result explained the effect by demonstrating enhanced cellular internalization mediated by LPN vehicle and iRGD decoration. In addition, we adopted the combination of single PTX/iRGD LPN and free TET (P/iRGD LPN+T) to evaluate the advantages of co-loading drugs. Although both P/iRGD LPN+T and P+T/iRGD LPN contained the same amount of TET, the P-gp inhibition efficiency of P+T/iRGD LPN was much higher than that of P/iRGD LPN+T. This could be attributed by the different cellular internalization capacity of TET in free phase and co-loaded in LPN. TET loaded in LPN was more likely to be taken up by cells than free TET, which is the characteristic advantage of nanoparticles. Therefore, the enhanced uptake and P-gp inhibition of P+T/iRGD LPN resulted in intensive cytotoxicity, ROS production, apoptosis and mitosis arrest by microtubule depolymerization of A2780/PTX cells. Meanwhile, the therapeutic efficacy of the combination of PTX/LPN and free TET was not significantly higher than that of free drugs combination. Therefore, we concluded that the enhanced suppression efficacy of A2780/PTX cells was ascribed to the co-loading of PTX and TET in iRGD functionalized LPN with a sequential drug-release profile.

To selectively transport drugs to tumor through active-targeting nanoparticles would be a useful approach to improve the MDR reversal activity and minimize side-effects. Compared to non-specific nano-carriers or free drugs, drugs loaded in nanoparticles modified with tumor-targeting ligands can enter the target cells through different mechanisms to avoid the drug transporter P-gp, MRP and MCRP and to increase the intracellular accumulation of drugs, so that to reverse MDR. Thus, to implement the tumor targeting is a prerequisite. In this study, we observed iRGD ligands grafted on LPNs could remarkably enhance the cellular uptake in both A2780 and A2780/PTX cells, indicated that iRGD peptide could improve drugs internalization. However, the *in vivo* study on the tumor tissue distribution and penetration of iRGD modified LPNs should be carried out in future work. More importantly, the significant MDR overcoming efficacy of iRGD-LPN with PTX and TET co-loading should be investigated in tumor bearing mice model. Moreover, although drugs combination and nanomaterials for drug delivery strategy have been applied increasing widely in cancer treatment, the potential toxicity and side-effect on organism has not yet caught up with the rapid application. Using high-throughput or systems biology methods like metabonomics would hold excellent prospect for tumor treatment research in the future.

In summary, a multifunctional lipid-polymer nanoparticle (LPN) system with codelivery of PTX and TET was developed to overcome MDR in a programmed manner. Primarily, the incorporation of iRGD peptide on LPN resulted in greater cancer cell targeting and penetration effects. After integrin receptor-mediated endocytosis, the loaded TET was spontaneously and rapidly released to inhibit the P-gp pump. PTX conjugated to the polymeric core was then redox-sensitively released into the cytoplasm and greatly accumulated in the cells. As a proof of concept, iRGD-functionalized LPN of both PTX and TET codelivery demonstrated significant drug loading efficiency, stability, cytotoxicity and proapoptotic activity against A2780/PTX cells. Our study provides an effective and robust strategy for drug co-delivery to significantly overcome tumor drug resistance.

## Method

### Materials

Paclitaxel (PTX, purity ≥98%) and tetrandrine (TET, purity ≥98%) were purchased from Melonepharma Co., Ltd. (Dalian, China). MAL-PEG-DSPE (1,2-distearoyl-sn-glycero-3-phosphoethanolamine-N-[maleimide(polyethylene glycol)-2000]) was purchased from Avanti Polar Lipids, Inc. (Alabaster, USA). PLGA (D,L-lactide-co-glycolide, MW 5000, lactide: glycolide (50:50)), dicyclohexylcarbodiimide (DCC), 4-dimethylaminopyridine (DMAP), 3,3′-dithiodipropionic acid (DTPA), egg lecithin (EPC), cholesterol (CHOL), MTT and Hoechst 33342 were obtained from Sigma-Aldrich (St. Louis, MO). iRGD peptide (sequence: CRGDK/RGPD/EC, purity >95%) with a residual thiol group was custom-synthesized by GL Biochem Co. Ltd. (Shanghai, China). All other chemicals were of analytical grade and were used as received.

The sensitive human ovarian carcinoma A2780 and paclitaxel-resistant A2780/PTX cell lines were obtained from KeyGen Biotech Co., Ltd. (Nanjing, China) and were maintained in DMEM (Life Technologies Co.) containing 10% fetal bovine serum (FBS, Gibco), 2 mM L-glutamine, penicillin-streptomycin solution (40 U/mL each, Gibco, Life Technologies Co.) at 37 °C in a humidified CO_2_ (5%) incubator.

### Synthesis and characterization of PLGA-SS-PTX conjugate

To decrease the by-products of PLGA-SS-PLGA, 3,3′-dithiodipropionic acid anhydride (DTPAA) was obtained in advance by the cyclization reaction between DTPA and acetyl chloride at 65 °C for 2 h^49^, to replace the linear DTPA. PLGA-DTPA conjugates were subsequently synthesized by a ring-opening reaction of PLGA with DTPAA. Briefly, PLGA (0.25 mmol) and DTPPA (0.3 mmol) were dissolved in 10 mL of anhydrous DMSO. Then, 0.3 mmol of triethylamine (TEA) was added, and the mixture was reacted at 40 °C for 8 h. The PLGA-DTPA crude product was obtained by successively dialyzing against DMSO and deionized water using a dialysis tube (MWCO 1000) before being lyophilized.

The disulfide-containing PLGA-SS-PTX conjugate was synthesized through an esterification reaction between PTX and the terminal carboxyl in PLGA-DTPA using DCC and DMAP as catalysts. Briefly, PLGA-DTPA (0.2 mmol), PTX (0.3 mmol), DCC (0.5 mmol), and DMAP (0.5 mmol) were dissolved together in 20 mL of anhydrous DMSO. The reaction mixture was maintained with mild stirring at 40 °C for 24 h. Similarly, PLGA-SS-PTX crude product was obtained by successively dialyzing against DMSO and deionized water using a dialysis tube (MWCO 1000) to remove by-products such as dicyclohexylurea and unreacted small molecules. The purified PLGA-SS-PTX was obtained by freeze-drying and was kept at 4 °C for use. ^1^H NMR of the PLGA-SS-PTX conjugate was performed using deuterated DMSO as a solvent. The PTX loading efficiency in this conjugate was determined by an HPLC method at 227 nm with acetonitrile/water (70/30, v/v) as the mobile phase.

### Optimization of drug ratio

A2780 and A2780/PTX cells were seeded into 96-well plates at a density of 5 × 10^3^ cells/well. After being cultured for 24 h, the cells were treated with PTX alone, TET alone; or the combination of the two drugs at molar ratios of 1/0.5, 1/1, 1/1.5 or 1/2 (PTX/TET). After 48-h incubation, cell viability was assessed by MTT assay. The data was presented as the mean and standard deviation of 6 replicates.

The reversal index of TET was calculated from the ratio of IC_50,PTX_ and IC_50,P/T_. The combination index (CI) value was calculated based on the additive effect of the two drugs using the median-effect analysis shown in Equation (1)^50^:


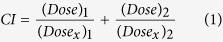


Where (Dose)_1_ and (Dose)_2_ are the concentrations of drug 1 and drug 2, respectively, in the combination that inhibits *x*% cells and (Dose_*x*_)_1_ and (Dose_*x*_)_2_ are the concentrations of drug 1 and drug 2, respectively, as a single drug that inhibits *x*% cells. The interaction of the two drugs can be classified as synergistic (CI < 1), additive (CI = 1) or antagonistic (CI > 1)^51^.

### Preparation of PTX+TET co-loaded LPN

The LPNs were prepared by a single-step method to synchronize a nanoprecipitation process with a simultaneous self-assembly process^52^. In brief, EPC/CHOL/MAL-PEG-DSPE (molar ratio = 1:1:8), at a weight ratio of 20% to the PLGA-SS-PTX conjugate, was dissolved in EtOH and added to water at 65 °C under stirring. Subsequently, the PLGA-SS-PTX conjugate and TET (PTX: TET = 1:1.2, molar ratio) were dissolved in ACN at 5 mg/mL and added dropwise to the heated lipid solution with gentle stirring for another 2 hours to allow the organic solvent to evaporate. Organic solvent residue in the LPN solution was finally removed by vacuum rotary evaporation at 40 °C. The LPNs were obtained to remove unloaded molecules by 0.45-μm filter filtration.

A post-insertion method was employed to conjugate iRGD peptide onto preformed robust LPNs based on the effortless binding between the thiol in iRGD peptide and the maleimide group on the LPN surface. Various ratios of iRGD peptide and MAL-PEG-DSPE polymer were added to and incubated with LPNs for 1 h at room temperature in an inert N_2_ atmosphere. The excess free iRGD peptides were removed by ultrafiltration (MWCO 3500 Da). The concentration of decorated iRGD on the LPNs was determined by an HPLC method at 220 nm with acetonitrile and water containing 0.1% TFA (V/V = 40:60) as the mobile phase.

### Characterization of LPNs

The particle size, polydispersity index and zeta potential of three batches of LPNs were determined by dynamic light scattering (DLS, Malvern Zetasizer Nano ZSP) at 25 °C. The stability of the LPNs in PBS (pH 7.4) with or without 10% FBS at 4 °C was evaluated. The LPN image was analyzed by a Tecnai G20 transmission electron microscope (TEM, FEI, Co., USA) at an operation voltage of 200 kV.

To measure the encapsulation efficiency (EE) and loading efficiency (LE) of TET, the LPNs were dissolved in methanol to disrupt the polymeric shells before HPLC analysis. Then, the TET concentration was determined using a Waters e2695 HPLC equipped with a reverse phase C_18_ column (150 × 4.6 mm, 5 μm) at a maximum absorbance of 280 nm. The mobile phase was MeOH/0.03% TFA buffer (80/20, v/v) at a flow rate of 1 mL/min. The EE and LE were calculated using the following equations (2, 3), respectively:


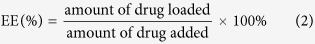



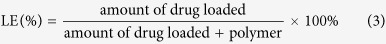


### *In vitro* release of co-loaded LPN

The release of PTX and TET from the iRGD-LPNs was analyzed using dialysis. The free drug combination at an optimized ratio dissolved in a Cremophor EL and ethanol (1:1, v/v) mixed solvent was used as a control. Briefly, a 2-mL LPN suspension or free drug mixture was transferred into a dialysis tube with MWCO 7,000 and was dialyzed against 40 mL phosphate buffer at pH 7.4 with or without 10 mM glutathione (GSH) under stirring at 100 rpm. At predetermined periods (0.5, 1, 2, 4, 8, 12, 24, 48, 72, 96 h), 1 mL of dialysis medium was sampled for PTX and TET measurement by HPLC using the aforementioned protocols. Meanwhile, 1 mL of fresh medium was added back to the dialysis medium after each sampling to maintain a constant volume. All drug release experiments were performed three times.

### Cellular uptake of co-loaded LPNs

To determine the cellular accumulation of PTX mediated by P-gp inhibition due to TET and iRGD LPN incorporation, the A2780/PTX cell uptake of PTX in various formulations was determined by HPLC. A2780/PTX cells (2.0 × 10^4^/well) were plated into 6-well plates and incubated for 24 h to allow attachment. Various formulations, including free PTX, free P + T, P + T/LPN, and P + T/iRGD LPN, with equivalent concentrations of both PTX and TET at 20 μM were added to the cells. At 1, 2, and 4 h post-incubation at 37 °C, the cells were washed twice with cold PBS to remove drugs from the cell surface, collected and resuspended in 0.2 mL of RIPA lysis buffer (Beyotime, China). After 20 min of cell-liquid extraction at 4 °C, the cell lysate was obtained by centrifugation at 12000 rpm for 15 min. PTX in the cell lysate was extracted by ultrasound with an equal volume of acetonitrile. The supernatant was removed for HPLC analysis. The amount of total proteins in the cell lysate was also determined using the Bradford method (Bio-Rad, USA). The intracellular uptake of PTX was determined as the PTX content normalized to the cellular protein content.

### Rhodamine 123 accumulation in A2780/PTX cells

To visualize the suppression effect of TET on the transport activity of P-gp, Rho 123, a fluorescent P-gp substrate, was employed to replace PTX. The PLGA-SS-Rho 123 conjugate was synthesized based on the synthesis route of the PLGA-SS-PTX conjugate with a slight modification. The Rho 123 content was determined by HPLC at 500 nm with a mobile phase consisting of acetonitrile, 20 mM sodium acetate buffer pH 4.0, and water containing 1.5 mM TBA (50:20:30)^53^.

Briefly, A2780/PTX cells (2 × 10^4^ cells/well) were treated with free Rho 123 (10 μM), free R + T (10 μM), R + T/LPN, or R + T/iRGD LPN for 4 h. Then, the culture media was replaced with free media and incubated for another 1 h. At the end of incubation, the cells were washed three times with PBS to remove free Rho 123, followed by fixation with 4% paraformaldehyde and staining by Hoechst 33342. The cellular accumulation of Rho 123 in the various treatment groups was observed using an Incell 2000 Analyzer (GE, Buckinghamshire, UK).

### P-gp inhibition and *in vitro* cytotoxicity study

To evaluate whether the observed cytotoxicity was related to P-gp inhibition effects, A2780/PTX cells were pretreated with various samples containing PTX and TET. Briefly, A2780 and A2780/PTX cells (5.0 × 10^4^ cells/well) were seeded into 6-well plates separately and incubated for 24 h. Then, the cells were treated with free PTX, free TET, free PTX+TET, P/LPN+T, P/iRGD LPN+T, P+T/LPN, or P+T/iRGD LPN at equivalent concentrations of both PTX and TET at 10 μM for 4 h. At the end of incubation, 1 μL of P-gp antibody (FITC) was added to the cells and incubated for another 1 h. After collection by trypsin and a PBS wash, the A2780 and A2780/PTX cells were detected using flow cytometry (FCM). The obtained values were expressed as relative intensity compared to the untreated controls.

Cell viability determination was performed as previously described. After being seeded in a 96-well plate for 24 h, A2780/PTX cells (5.0 × 10^3^ cells/well) were treated with various samples as mentioned above. After 48 h and 72 h incubation, the cells were incubated with 1 mg/mL MTT medium for another 4 h, and then the formazan crystals were dissolved with DMSO. The relative cell viability compared to the untreated control was determined by detecting the 570 nm spectrophotometric absorbance with a microplate reader.

### Intracellular ROS determination

The intracellular ROS levels was detected by the fluorescent probe 5-(and-6)-chloromethyl-2′,7′-dichlorodihydrofluorescein diacetate (DCFH-DA, Life Technologies). A2780/ADR cells (5.0 × 10^4^ cells/well) were seeded in 6-well plates and allowed to attach for 24 h. After being exposed to blank LPN (250 μg/mL), free PTX+TET, P/LPN+T, P/iRGD LPN+T, P+T/LPN, or P+T/iRGD LPN at equivalent concentrations of both PTX and TET at 10 μM for 24 h, the cells were stained by 1 μM DCFH-DA for 30 min at 37 °C. After washing the cells three times with cold PBS, the fluorescence intensity was detected using a flow cytometer (FCM, BD Biosciences, California, USA) at an emission wavelength of 525 nm and an excitation wavelength of 488 nm. The obtained values were expressed as relative intensity compared to the untreated controls.

### Apoptosis analysis

Apoptosis was detected with an Annexin V-FITC/PI detection kit (Biovision, USA). A2780/PTX cells (5.0 × 10^4^ cells/well) were seeded in 6-well plates and treated with blank LPN (250 μg/mL), free PTX+TET, P/LPN+T, P/iRGD LPN+T, P+T/LPN, or P+T/iRGD LPN at equivalent concentrations of both PTX and TET at 10 μM for 48 h. After collected with trypsin, the cells were gently washed with PBS and resuspended in 100 μL binding buffer, which contained 5 μL of PI solution and 3 μL of Annexin V-FITC, for 15 min. Finally, each sample was analyzed by using a flow cytometer (BD Biosciences, California, USA).

### Cell cycle analysis

A2780/PTX cells (5.0 × 10^4^ cells/well) were seeded in 6-well plates and treated with blank LPN (250 μg/mL), free PTX+TET, P/LPN+T, P/iRGD LPN + T, P+T/LPN, or P+T/iRGD LPN at equivalent concentrations of both PTX and TET at 10 μM for 48 h. The cells were harvested and washed with PBS and were then fixed in cold 70% ethanol and stored at −20 °C overnight. The cells were collected by centrifugation and stained with 5 μL of PI solution (Life Technologies, USA) for 10 min. The cell cycle results were obtained using flow cytometry (BD Biosciences, California, USA), and the cell distributions were analyzed using ModFit LT software (version 3.0, USA).

### Caspase-3/7 activity determination assay

The caspase-3/7 assay kit (Promega Kit #G8091) consisted of a substrate that produces fluorescent rhodamine when cleaved by caspases-3/7. Briefly, A2780/PTX cells (5.0 × 10^4^ cells/well) were seeded in 6-well plates and treated with blank LPN (250 μg/mL), free PTX+TET, P/LPN+T, P/iRGD LPN+T, P+T/LPN, or P+T/iRGD LPN at equivalent concentrations of both PTX and TET at 10 μM for 48 h. Afterwards, the cells were collected and resuspended in caspase buffer with substrate for 2 h. Caspase-3/7 activity was determined from the fluorescence produced by apoptosis cells at an excitation wavelength of 485 nm and an emission wavelength of 527 nm. The obtained values were expressed as fluorescence intensity relative to the controls.

### Tubulin immunofluorescence analysis

For the immunofluorescence assay, A2780/PTX cells (5.0 × 10^3^ cells/well) were seeded in 96-well plates and then treated with free PTX, free PTX+TET, P+T/LPN, or P+T/iRGD LPN at equivalent concentrations of both PTX and TET at 10 μM for 24 h. After being washed with PBS three times and hydrated for 1 h, the cells were fixed in 4% PFA for 1 h and washed twice with PBS. Subsequently, the cell nuclei were stained using a Hoechst 33342 solution (1 μg/mL) for 10 min, and then the cells were hydrated with PBS for 1 h. After permeabilized for 20 min with 0.5% Triton X-100 buffer, the cells were blocked for 1 h using 0.2% Triton X-100 buffer containing 5% BSA. α-tubulin-FITC solution (dilution ratio 1:100) was added into each well for 8 h incubation at 4 °C. Afterwards, the cells were resuspended with PBS and observed using an Incell 2000 Analyzer.

### Western-blot analysis

A2780/PTX cells were cultured under the same conditions as the *in vitro* cytotoxicity studies. Likewise, various formulations including free PTX+TET, P/LPN+T, P/iRGD LPN+T, P+T/LPN, and P+T/iRGD LPN with equivalent concentrations of both PTX and TET at 10 μM were employed to treat cells for 24 h. Subsequently, cell lysates were prepared, electrotransferred on 6 cm of 10% polyacrylamide gels, blocked, and then immunoblotted with primary antibodies: Akt, p-Akt, Bcl-2 and Bax (Cell Signaling Technology, USA). Anti-rabbit GAPDH antibody was used as internal reference. The membrane was incubated with horseradish peroxidase-conjugated secondary antibodies for 1 h and visualized using an ECL advanced Western blotting detection kit. Photos of protein bands were taken by a Molecular Imager ChemiDoc XRS (Bio-rad). Densitometric measurements of band intensity in the Western blots were performed using Quantity One Software. The results were expressed as relative protein levels compared to the relative total protein ± SD of three independent experiments.

### Statistical analysis

All data are presented as mean ± SD. One-way ANOVA was performed to determine statistical differences and p < 0.05 was considered to be significant.

## Additional Information

**How to cite this article:** Zhang, J. *et al*. Co-delivery of paclitaxel and tetrandrine via iRGD peptide conjugated lipid-polymer hybrid nanoparticles overcome multidrug resistance in cancer cells. *Sci. Rep.* **7**, 46057; doi: 10.1038/srep46057 (2017).

**Publisher's note:** Springer Nature remains neutral with regard to jurisdictional claims in published maps and institutional affiliations.

## Figures and Tables

**Table 1:** Cytotoxicity of PTX, TET, and PTX/TET to PTX-sensitive A2780 cells and PTX-resistant A2780/PTX cells after 48 h treatments (n = 3).

Cells	Drugs	IC_50_ values (μM)	Reversal index	CI values at IC_50_
A2780	PTX	1.78	—	
A2780/PTX	PTX	86.4	—	
	TET	45.1	—	
	P/T (1/0.5)	28.5/14.25	3.03	0.64
	P/T (1/1)	14.7/14.7	5.87	0.49
	P/T (1/1.5)	11.6/17.4	7.44	0.52
	P/T (1/2)	10.8/21.6	8	0.60

**Table 2:** Characteristic properties of LPNs.

Samples	Size (nm)	PDI	Potential (mV)	EE (%)	LE(%)	
				TET	PTX	TET
P/non-iRGD LPN	107.3 ± 8.6	0.16 ± 0.03	−18.2 ± 3.3	—	10.1 ± 0.49	—
P/iRGD LPN	110.1 ± 10.8	0.16 ± 0.04	−17.7 ± 3.1	—	11.6 ± 0.85	—
P + T/non-iRGD LPN	128.5 ± 11.3	0.18 ± 0.04	−16.8 ± 4.3	84.5 ± 6.8	10.4 ± 0.64	7.38 ± 0.58
P + T/iRGD LPN	139.5 ± 12.4	0.13 ± 0.02	−17.2 ± 2.4	82.8 ± 5.4	10.2 ± 0.38	7.46 ± 0.47

## References

[b1] Marcus C. S., Maxwell G. L., Darcy K. M., Hamilton C. A. & McGuire W. P. Current approaches and challenges in managing and monitoring treatment response in ovarian cancer. Journal of Cancer. 5, 25 (2014). 24396495 10.7150/jca.7810 PMC3881218

[b2] Bader A. Mechanisms of multidrug resistance in cancer. Cancer research (2014).

[b3] Saneja A., Dhar Dubey R., Alam N., Khare V. & N Gupta P. Co-formulation of P-glycoprotein substrate and inhibitor in nanocarriers: An emerging strategy for cancer chemotherapy. Current cancer drug targets. 14, 419–433 (2014). 24720364 10.2174/1568009614666140407112034

[b4] Saneja A., Khare V., Alam N., Dubey R. D. & Gupta P. N. Advances in P-glycoprotein-based approaches for delivering anticancer drugs: pharmacokinetic perspective and clinical relevance. Expert opinion on drug delivery. 11, 121–138 (2014). 24295039 10.1517/17425247.2014.865014

[b5] Fu L. . Characterization of tetrandrine, a potent inhibitor of P-glycoprotein-mediated multidrug resistance. Cancer chemotherapy and pharmacology. 53, 349–356 (2004). 14666379 10.1007/s00280-003-0742-5

[b6] Meng L.-h. . Tetrandrine induces early G1 arrest in human colon carcinoma cells by down-regulating the activity and inducing the degradation of G1-S–specific cyclin-dependent kinases and by inducing p53 and p21Cip1. Cancer research. 64, 9086–9092 (2004). 15604277 10.1158/0008-5472.CAN-04-0313

[b7] Liu B. . Anticancer effect of tetrandrine on primary cancer cells isolated from ascites and pleural fluids. Cancer letters. 268, 166–175 (2008). 18495333 10.1016/j.canlet.2008.03.059

[b8] Wang G., Lemos J. R. & Iadecola C. Herbal alkaloid tetrandrine: from an ion channel blocker to inhibitor of tumor proliferation. Trends in pharmacological sciences. 25, 120–123 (2004). 15058281 10.1016/j.tips.2004.01.009

[b9] Wan J. . Synergistic antitumour activity of sorafenib in combination with tetrandrine is mediated by reactive oxygen species (ROS)/Akt signaling. British journal of cancer. 109, 342–350 (2013). 23807172 10.1038/bjc.2013.334 PMC3721403

[b10] Zhang Y. . Combination of Tetrandrine with cisplatin enhances cytotoxicity through growth suppression and apoptosis in ovarian cancer in vitro and in vivo. Cancer letters. 304, 21–32 (2011). 21333438 10.1016/j.canlet.2011.01.022

[b11] Wei J. . Synergistic interaction between tetrandrine and chemotherapeutic agents and influence of tetrandrine on chemotherapeutic agent-associated genes in human gastric cancer cell lines. Cancer chemotherapy and pharmacology. 60, 703–711 (2007). 17256130 10.1007/s00280-007-0416-9

[b12] Li X. . Paclitaxel/tetrandrine coloaded nanoparticles effectively promote the apoptosis of gastric cancer cells based on “oxidation therapy”. Molecular pharmaceutics. 9, 222–229 (2011). 22171565 10.1021/mp2002736

[b13] Zhu X., Sui M. & Fan W. *In vitro* and in vivo characterizations of tetrandrine on the reversal of P-glycoprotein-mediated drug resistance to paclitaxel. Anticancer research. 25, 1953–1962 (2005). 16158930

[b14] Greco F. & Vicent M. J. Combination therapy: Opportunities and challenges for polymer-drug conjugates as anticancer nanomedicines. Adv Drug Deliver Rev. 61, 1203–1213, doi: 10.1016/j.addr.2009.05.006 (2009). 19699247

[b15] Kunjachan S., Rychlik B., Storm G., Kiessling F. & Lammers T. Multidrug resistance: Physiological principles and nanomedical solutions. Adv Drug Deliver Rev. 65, 1852–1865 (2013). 10.1016/j.addr.2013.09.018 PMC3939439 24120954

[b16] Ma Y. . Combinational delivery of hydrophobic and hydrophilic anticancer drugs in single nanoemulsions to treat MDR in cancer. Molecular pharmaceutics. 11, 2623–2630 (2014). 24712391 10.1021/mp400778r PMC4144753

[b17] Kirtane A. R., Kalscheuer S. M. & Panyam J. Exploiting nanotechnology to overcome tumor drug resistance: challenges and opportunities. Adv Drug Deliver Rev. 65, 1731–1747 (2013). 10.1016/j.addr.2013.09.001 PMC3849460 24036273

[b18] Ma L., Kohli M. & Smith A. Nanoparticles for combination drug therapy. ACS nano. 7, 9518–9525 (2013). 24274814 10.1021/nn405674m PMC3894659

[b19] Patil Y., Sadhukha T., Ma L. & Panyam J. Nanoparticle-mediated simultaneous and targeted delivery of paclitaxel and tariquidar overcomes tumor drug resistance. Journal of Controlled Release. 136, 21–29 (2009). 19331851 10.1016/j.jconrel.2009.01.021

[b20] Godsey M. E., Suryaprakash S. & Leong K. W. Materials innovation for co-delivery of diverse therapeutic cargos. RSC advances. 3, 24794–24811 (2013). 24818000 10.1039/C3RA43094D PMC4012692

[b21] Jabr-Milane L. S., van Vlerken L. E., Yadav S. & Amiji M. M. Multi-functional nanocarriers to overcome tumor drug resistance. Cancer treatment reviews. 34, 592–602 (2008). 18538481 10.1016/j.ctrv.2008.04.003 PMC2585991

[b22] Shapira A., Livney Y. D., Broxterman H. J. & Assaraf Y. G. Nanomedicine for targeted cancer therapy: towards the overcoming of drug resistance. Drug resistance updates. 14, 150–163 (2011). 21330184 10.1016/j.drup.2011.01.003

[b23] Sugahara K. N. . Tissue-penetrating delivery of compounds and nanoparticles into tumors. Cancer cell. 16, 510–520 (2009). 19962669 10.1016/j.ccr.2009.10.013 PMC2791543

[b24] Alberici L. . De novo design of a tumor-penetrating peptide. Cancer research. 73, 804–812 (2013). 23151901 10.1158/0008-5472.CAN-12-1668 PMC3548935

[b25] Duan X. . Smart pH-sensitive and temporal-controlled polymeric micelles for effective combination therapy of doxorubicin and disulfiram. ACS nano. 7, 5858–5869 (2013). 23734880 10.1021/nn4010796

[b26] Fan L. . Co-delivery of PDTC and doxorubicin by multifunctional micellar nanoparticles to achieve active targeted drug delivery and overcome multidrug resistance. Biomaterials. 31, 5634–5642 (2010). 20430433 10.1016/j.biomaterials.2010.03.066

[b27] Wang H. . Enhanced anti-tumor efficacy by co-delivery of doxorubicin and paclitaxel with amphiphilic methoxy PEG-PLGA copolymer nanoparticles. Biomaterials. 32, 8281–8290 (2011). 21807411 10.1016/j.biomaterials.2011.07.032

[b28] Patel N. R., Rathi A., Mongayt D. & Torchilin V. P. Reversal of multidrug resistance by co-delivery of tariquidar (XR9576) and paclitaxel using long-circulating liposomes. International journal of pharmaceutics. 416, 296–299 (2011). 21703341 10.1016/j.ijpharm.2011.05.082 PMC3156341

[b29] Clancy P. Nanoparticles: Self-assembly finds its own limits. Nature nanotechnology. 6, 540–541 (2011). 10.1038/nnano.2011.152 21897385

[b30] Li C. & Wallace S. Polymer-drug conjugates: recent development in clinical oncology. Adv Drug Deliver Rev. 60, 886–898 (2008). 10.1016/j.addr.2007.11.009 PMC2432086 18374448

[b31] Zhang L. . Self-assembled lipid− polymer hybrid nanoparticles: a robust drug delivery platform. ACS nano. 2, 1696–1702 (2008). 19206374 10.1021/nn800275r PMC4477795

[b32] Bao Y. . d-α-Tocopherol Polyethylene Glycol Succinate-Based Redox-Sensitive Paclitaxel Prodrug for Overcoming Multidrug Resistance in Cancer Cells. Molecular pharmaceutics. 11, 3196–3209 (2014). 25102234 10.1021/mp500384d

[b33] Lv S. . Well-defined polymer-drug conjugate engineered with redox and pH-sensitive release mechanism for efficient delivery of paclitaxel. Journal of Controlled Release. 194, 220–227 (2014). 25220162 10.1016/j.jconrel.2014.09.009

[b34] Matsumura Y. Poly (amino acid) micelle nanocarriers in preclinical and clinical studies. Adv Drug Deliver Rev. 60, 899–914 (2008). 10.1016/j.addr.2007.11.010 18406004

[b35] Laurent A. . Controlling tumor growth by modulating endogenous production of reactive oxygen species. Cancer research. 65, 948–956 (2005). 15705895

[b36] Elizabeth Fox B. C. W., Pastakia Devang, Chen Clara C., Yang Sherry X., Diane Cole & FrankBalis M.. Pharmacokinetic and pharmacodynamic study of tariquidar (XR9576), a P-glycoprotein inhibitor, in combination with doxorubicin, vinorelbine, or docetaxel in children and adolescents with refractory solid tumors. Cancer Chemother Pharmacol. 76, 1273–1283 (2015). 26486517 10.1007/s00280-015-2845-1 PMC7751951

[b37] Xiao W. . Tetrandrine induces G1/S cell cycle arrest through the ROS/Akt pathway in EOMA cells and inhibits angiogenesis *in vivo*. International journal of oncology. 46, 360–368 (2015). 25355542 10.3892/ijo.2014.2735

[b38] Sampath D. . MAC-321, a novel taxane with greater efficacy than paclitaxel and docetaxel *in vitro* and *in vivo*. Molecular cancer therapeutics. 2, 873–884 (2003). 14555706

[b39] Orr G. A., Verdier-Pinard P., McDaid H. & Horwitz S. B. Mechanisms of Taxol resistance related to microtubules. Oncogene. 22, 7280–7295, doi: 10.1038/sj.onc.1206934 (2003). 14576838 PMC4039039

[b40] Yusuf R. Z., Duan Z., Lamendola D. E., Penson R. T. & Seiden M. V. Paclitaxel resistance: molecular mechanisms and pharmacologic manipulation. Curr Cancer Drug Targets. 3, 1–19 (2003). 12570657 10.2174/1568009033333754

[b41] Satsangi A. . Synthesis of a novel, sequentially active-targeted drug delivery nanoplatform for breast cancer therapy. Biomaterials. 59, 88–101, doi: 10.1016/j.biomaterials.2015.03.039 (2015). 25956854

[b42] Wang Z. & Ho P. C. Self-assembled core-shell vascular-targeted nanocapsules for temporal antivasculature and anticancer activities. Small. 6, 2576–2583, doi: 10.1002/smll.201001122 (2010). 20976704

[b43] Ye L. . A pH-sensitive binary drug delivery system based on poly(caprolactone)-heparin conjugates. Journal of biomedical materials research. Part A. 102, 880–889, doi: 10.1002/jbm.a.34735 (2014). 23554308

[b44] Lv S. . Co-delivery of doxorubicin and paclitaxel by PEG-polypeptide nanovehicle for the treatment of non-small cell lung cancer. Biomaterials. 35, 6118–6129, doi: 10.1016/j.biomaterials.2014.04.034 (2014). 24794923

[b45] Gu G. . The influence of the penetrating peptide iRGD on the effect of paclitaxel-loaded MT1-AF7p-conjugated nanoparticles on glioma cells. Biomaterials. 34, 5138–5148 (2013). 23582684 10.1016/j.biomaterials.2013.03.036

[b46] Shen J. . iRGD Conjugated TPGS mediates codelivery of paclitaxel and survivin shRNA for the reversal of lung cancer resistance. Molecular pharmaceutics. 11, 2579–2591 (2013). 24236909 10.1021/mp400576f

[b47] Zhu Z. . The effect of hydrophilic chain length and iRGD on drug delivery from poly (ε-caprolactone)-poly (N-vinylpyrrolidone) nanoparticles. Biomaterials. 32, 9525–9535 (2011). 21903260 10.1016/j.biomaterials.2011.08.072

[b48] Perche F. & Torchilin V. P. Recent trends in multifunctional liposomal nanocarriers for enhanced tumor targeting. Journal of drug delivery. 2013, 705265, doi: 10.1155/2013/705265 (2013). 23533772 PMC3606784

[b49] Jia L. . Redox-responsive catiomer based on PEG-ss-chitosan oligosaccharide-ss-polyethylenimine copolymer for effective gene delivery. Polym. Chem. 4, 156–165 (2012).

[b50] Bijnsdorp I. V., Giovannetti E. & Peters G. J. Analysis of drug interactions. Methods Mol Biol. 731, 421–434, doi: 10.1007/978-1-61779-080-5_34 (2011). 21516426

[b51] Duong H. H. P. & Yung L. Y. L. Synergistic co-delivery of doxorubicin and paclitaxel using multi-functional micelles for cancer treatment. Int J Pharmaceut. 454, 486–495, doi: 10.1016/j.ijpharm.2013.06.017 (2013). 23792465

[b52] Mandal B. . Core–shell-type lipid–polymer hybrid nanoparticles as a drug delivery platform. Nanomedicine: Nanotechnology, Biology and Medicine. 9, 474–491 (2013). 10.1016/j.nano.2012.11.010 23261500

[b53] Iqbal T., Kinjo M. & Dowling T. C. Determination of Rhodamine 123 in cell lysate by HPLC with visible wavelength detection. Journal of Chromatography B. 814, 259–262 (2005). 10.1016/j.jchromb.2004.10.037 15639447

